# The impact of preoperative serum lactate dehydrogenase on mortality and morbidity after noncardiac surgery

**DOI:** 10.1038/s41598-024-53372-x

**Published:** 2024-03-28

**Authors:** Yingchao Zhu, Juan Xin, Yaodan Bi, Tao Zhu, Bin Liu

**Affiliations:** 1 Department of Anesthesiology, West China Hospital, Sichuan University, No.37, Guoxue Valley, Chengdu, 610000 Sichuan China; 2 Department of Anesthesiology, Peking Union Medical College Hospital, Peking Union Medical College and Chinese Academy of Medical Sciences, Beijing, China

**Keywords:** Noncardiac surgery, Postoperative outcomes, Serum lactate dehydrogenase, Serum biomarkers, Biomarkers, Diseases

## Abstract

Preoperative serum lactate dehydrogenase (LDH) has been reported to be associated with adverse outcomes following thoracic surgery. However, its association with outcomes in noncardiac surgery as a whole has not been investigated. We conducted a retrospective cohort study at West China Hospital, Sichuan University, from 2018 to 2020, including patients undergoing noncardiac surgery. Multivariable logistic regression and propensity score weighting were employed to assess the link between LDH levels and postoperative outcomes. Preoperative LDH was incorporated into four commonly used clinical models, and its discriminative ability, reclassification, and calibration were evaluated in comparison to models without LDH. Among 130,879 patients, higher preoperative LDH levels (cut-off: 220 U/L) were linked to increased in-hospital mortality (4.382% vs. 0.702%; OR 1.856, 95% CI 1.620–2.127, *P* < 0.001), myocardial injury after noncardiac surgery (MINS) (3.012% vs. 0.537%; OR 1.911, 95% CI 1.643–2.223, *P* < 0.001), and ICU admission (15.010% vs. 6.414%; OR 1.765, 95% CI 1.642–1.896, *P* < 0.001). The inverse probability of treatment-weighted estimation supported these results. Additionally, LDH contributed significantly to four surgical prognostic models, enhancing their predictive capability. Our study revealed a significant association between preoperative LDH and in-hospital mortality, MINS, and ICU admission following noncardiac surgery. Moreover, LDH provided supplementary predictive information, extending the utility of commonly used surgical prognostic scores.

## Introduction

Previously, some studies have focused on exploring risk factors for noncardiac postoperative outcomes using routine laboratory tests, including hydroxybutyrate dehydrogenase (HBDH), neutrophil-to-lymphocyte ratio (NLR), hemoglobin (HB), albumin (ALB), and others^1–6^. The research on these indicators aims to investigate their roles in the pathophysiological status and prognosis of postoperative patients, providing a more comprehensive risk assessment. However, despite some achievements in past studies, considering their limitations and the limited understanding of comprehensive pathophysiology, we believe it is necessary to expand the focus to the application of lactate dehydrogenase (LDH).

Compared to other indicators, LDH, as an enzyme marker, covers multiple biological processes, including cell damage, inflammation, metabolism, and immune regulation. These factors may trigger inflammatory reactions and worsen oxygenation after surgery, increasing the risk of death. This makes LDH provide more extensive and in-depth biological information, potentially more comprehensively reflecting the preoperative physiological status of patients. This study continues this research approach by delving into the application of LDH, aiming to provide a more comprehensive and accurate preoperative risk assessment for noncardiac surgical patients, as well as meaningful biomarkers for monitoring during and after surgery. This not only offers more decision support for clinical physicians but also has the potential to provide new directions and insights for future related research, advancing a deeper understanding of postoperative outcomes.

Nowadays, serum LDH examination has become part of the routine preoperative biochemical test. Preoperative high LDH is common, with the incidence ranging from 10 to 35%^7–9^. A study of 626 patients has shown that preoperative serum LDH level is an independent predictor of cardiopulmonary complications following thoracoscopic lobectomy or segmental resection^9^. However, in the context of noncardiac surgery, the relationship between LDH and morbidity and mortality following surgery has not been thoroughly investigated.

Therefore, we aimed to evaluate the relationship between preoperative serum LDH levels and prognosis after noncardiac surgery and to explore whether the relationship persisted after the propensity score weighting methods and subgroup analysis.

## Methods

### Study design and data collection

The Strengthening the Reporting of Observational Studies in Epidemiology^10^ declaration is followed by this retrospective cohort study. In-hospital deaths, ICU admission, and myocardial injury after noncardiac surgery (MINS) were documented with death certificates and medical record reviews. Due to the sensitive nature of the data used in this study, hospital information center staff members without knowledge collected the data. Independent researchers who were blind to the outcomes compiled the baseline features into a standardized form after obtaining the raw data from the preoperative evaluation sheets. Qualified researchers with experience in human subject confidentiality agreements carried out the data analysis. All data were anonymized and de-identified for confidentiality reasons. This study was approved by Ethics committee in September 2021 (Project No.1082 in 2021), and the need for informed consent was waived by Ethics committee (the Ethics Committee on Biomedical Research, West China Hospital of Sichuan University). The study was performed in accordance with the Declaration of Helsinki and registered at chictr.org (ChiCTR2300068425).

We screened all patients over 14 years old who underwent surgery in West China Hospital of Sichuan University from February 2018 to November 2020. The following patients were excluded: (1) People having ophthalmic, cardiac, obstetric, and diagnostic surgery. (2) patients who didn't have a preoperative LDH measurement available.

### Outcome

The primary outcome was in-hospital mortality, defined as all-cause mortality that occurred during postoperative hospitalization. The secondary outcomes included ICU admission and MINS. ICU admission was defined as patients who stayed in the ICU for more than 24 h and excluded patients who were in the ICU preoperatively. Patients who were in the ICU before surgery were excluded from ICU-related analyses. MINSis defined as high-sensitive postoperative troponin T (hs-cTnT) > 30 mmol/l, that occurs during or within 30 days after surgery.^11,12^ Clinicians screened high-risk groups for detection of myocardial injury, according to clinical guidelines and experience. Patients without a postoperative cardiac enzyme determination were assumed not to have an acute myocardial injury.

### LDH measurements and management

Serum LDH examination was a routine preoperative biochemical test for all surgical patients in our hospital. All serum LDH was measured by Lactate Dehydrogenase acc. to IFCC ver.2 (LDHI2) using the International Federation of Clinical Chemistry and laboratory medicine (IFCC) reference method in a Cobas 8000 Modular Autoanalyzer (Roche Diagnostics, Basel, Switzerland). Samples with hemolysis index > 15 were discarded. Preoperative LDH level was defined as the last measured serum LDH concentration within 3 days before surgery. The patient’s serum LDH ranged from 10 to 1,000 U/L. The high LDH group was defined as serum LDH > 220 U/L, according to the minimum positive value set by our laboratory.

### Statistical analysis

We examined the patient characteristics between normal and high LDH groups. The Mann–Whitney test or the t-test were used to compare differences in continuous data, which were provided as median with interquartile range. The χ2 or Fisher exact test was used to compare categorical data that were given as numbers (percentages).

The sample size is calculated according to the guidelines for a sample size of the clinical prediction model^13^. The highest R^2^ value at 1.09% mortality was 0.11. According to our conservative assumption, the new model will account for 15% of the variability; hence, the expected R^2^ value is 0.11 × 0.15 = 0.0165. The expected shrinkage required was set as a conservative 2.5%, to minimize the potential overfitting. The output shows that at least 82,026 samples are needed, which is numerically equal to 903 events and 26 events per covariate.

We constructed a multivariable logistic regression model to prove the association between the preoperative serum LDH level and outcomes after surgery. In addition to the well-established predictors^14^, we added as covariates factors that showed significant associations with mortality and morbidity in our preliminary experiments. Variables of skewness distribution were included in the model after logarithmic transformation. Then, least absolute shrinkage and selection operator regression (LASSO) was used to filter variables and adjust the complexity of the logistic regression model to reduce overfitting. Collinearity was evaluated by the variance inflation factor (VIF), and only variables with VIF ≤ 10 were input into the model. The variable selection process consisted of a preliminary variable selection using LASSO, which was then submitted to the clinicians for final confirmation to ensure that the final set of variables was statistically significant and clinically interpretable. The variables ultimately included in constructing the multivariable logistic regression model, after the selection process, encompass general patient conditions (hypertension, ischemic heart disease, diabetes, chronic obstructive pulmonary disease [COPD], liver disease, renal failure, malignancy), demographic factors (gender, age, Body Mass Index, preoperative heart rate, American Society of Anesthesiologists Physical Status [ASA-PS]), surgical characteristics (emergency, anesthesia method, surgery sites, anesthesia duration), and blood biomarkers (hemoglobin, white blood cell count, neutrophil-to-lymphocyte ratio, blood urea nitrogen, serum creatinine, serum albumin, alkaline phosphatase, serum globulin).

Similar to our another study^1^, we further analyzed the robustness of the association between different LDH levels and postoperative death using the treatment weighted inverse probability (IPTW)^15,16^ method. A standardized mean difference (SMD) of less than 10% was considered to be balanced between IPTW-matched groups. A restricted spline fitting curve was constructed to simulate the potential non-linear relationship between outcome and LDH.

‘Extended model’ was calculated by adding the preoperative LDH variable to the score of the four commonly used clinical models including American Society of Anesthesiologists (ASA), Charlson Comorbidity Index (CCI), Revised Cardiac Risk Index (RCRI), and Combined Assessment of Risk Encountered in Surgery (CARES)^17^. We explored the performance differences between extended models with or without preoperative LDH variables. The discrimination of the prediction models was assessed by the area under the receiver operating characteristic curve (AUROC)^18^. The reclassification power was assessed by the net reclassification improvement (NRI), and the Integrated Discrimination Increment (IDI). The calibration of the models was assessed using the Hosmere-Lemeshow goodness-of-fit test. We also use the Brier score to indicate overall model performance.

It was determined whether the association continued during subgroup analyses. We analyzed data separately for different sexes, age groups, ASA-PS scores, emergency case, anaesthesia methods and surgical sites, and with versus without comorbidities including hypertension, ischemic heart disease, diabetes, COPD, liver disease, renal failure, and malignancy tumor. In assessing the odds ratios (OR) for the association between serum LDH levels and post-surgery mortality, each subgroup was treated as independent data. The associated subgroup analysis excluded pertinent variables, while the logistic regression analysis proceeded with the inclusion of the remaining variables.

We conducted a sensitivity analysis by excluding individuals under 18 years of age. The primary analysis focused on ages over 14 years, but as a sensitivity check, we restricted the age criteria to individuals over 18 years.

R 4.0.2 (Vienna, Austria; http://www.R-project.org/) was used to conduct the statistical analyses.

### Ethics approval and consent to participate

This study was approved by Ethics committee in September 2021 (Project No.1082 in 2021), and the need for informed consent was waived by Ethics committee (the Ethics Committee on Biomedical Research, West China Hospital of Sichuan University).

## Results

### Baseline characteristics

**Figure 1:**
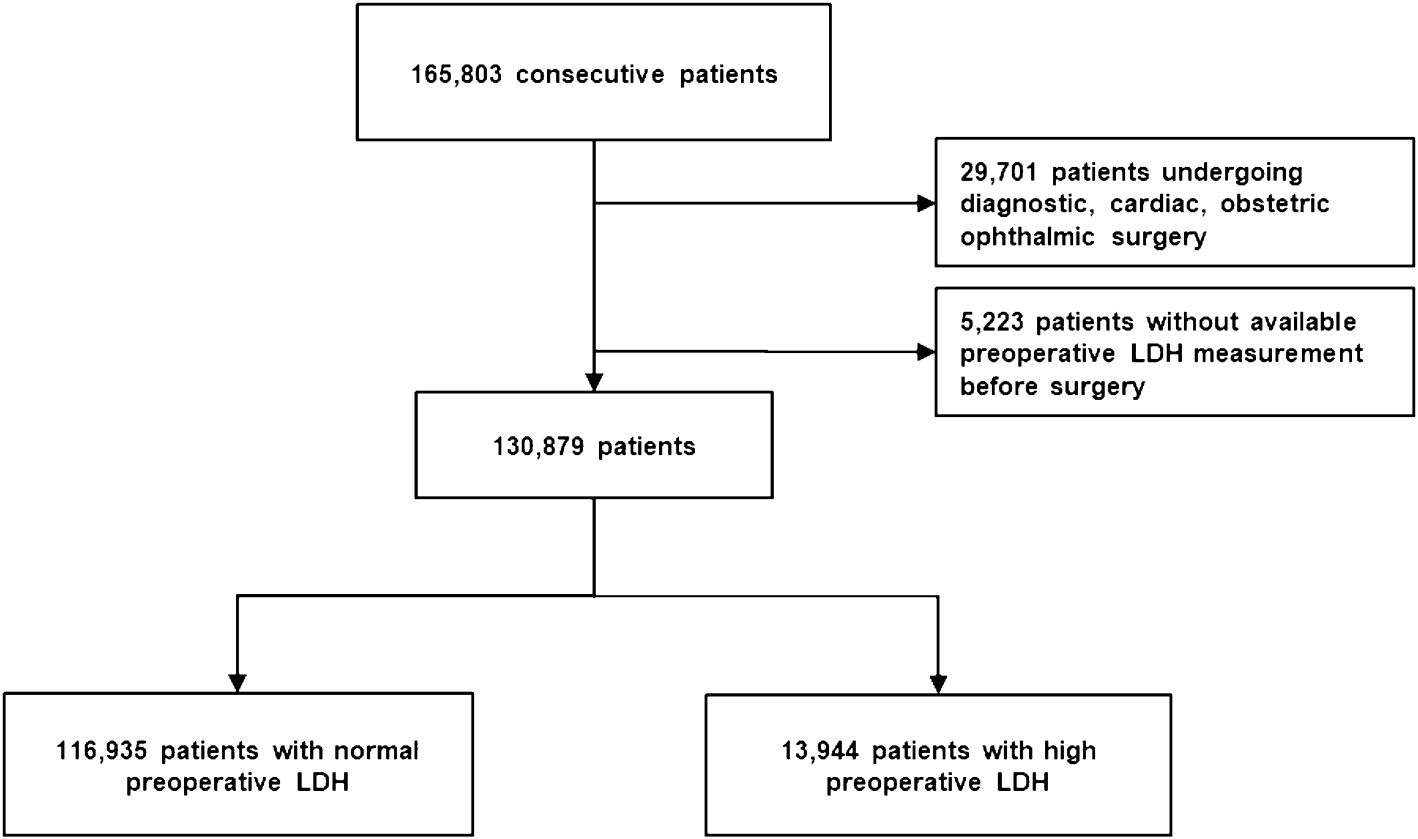
Patient flowchart.

Figure 1 depicts the patient flowchart. Our analysis included 130,879 patients, whose death, MINS, and ICU admission rates were, respectively, 1.109%, 0.801%, and 7.33%. Preoperative LDH values were taken in 96.16% of the total patients. Patients without LDH readings had rates of death, MINS, and ICU admission of 1.05%, 1.24%, and 6.49%, respectively. The rate between those who had LDH measurement and who did not was comparable. The median of LDH measurements was 164.0 (143.0–190.0) U/L.

There were 6193 patients who had hs-cTnT test postoperatively, of which 1048 patients had hs-cTnT higher than 30 mmol/l. Patients had a median age of 52.0 [40.0–63.0] years with females 64,311 (40.1%). Dividing the patients according to high and normal LDH, 13,944 patients (10.65%) had high preoperative LDH, and 116,935 (89.35%) had normal ones. Table 1 contrasts patients with normal LDH and those with high LDH in terms of demographics, preoperative factors, and perioperative characteristics. The prevalence of ischemic heart disease, COPD, and central nervous system (CNS) surgery was all higher in patients with high LDH.

**Table 1:** Baseline characteristics of patients, according to LDH level.

Variable	Overall	LDH < = 220	LDH > 220	*P*-value
n	130,879	116,935	13,944	
Sex (%)				
Men	66,561 (50.9)	59,222 (50.6)	7339 (52.6)	< 0.001
Women	64,318 (49.1)	57,713 (49.4)	6605 (47.4)	
Age (%)				
Age < = 60	91,352 (69.8)	82,632 (70.7)	8720 (62.5)	< 0.001
Age > 60	39,527 (30.2)	34,303 (29.3)	5224 (37.5)	
BMI (median [IQR]), kg/m^2^	23.00 [21.00, 25.00]	23.00 [21.00, 25.00]	23.00 [21.00, 26.00]	< 0.001
SBP (median [IQR]), mmHg	124 [113, 137]	124 [112, 137]	126 [114,140]	< 0.001
Heart rate (median [IQR]), Beats per minute	80 [73, 89]	80 [73, 89]	81 [74, 91]	< 0.001
ASA-PS (%)				
I–II	96,406 (73.7)	88,445 (75.6)	7961 (57.1)	< 0.001
III	32,818 (25.1)	27,625 (23.6)	5193 (37.2)	
IV–V	1655 (1.3)	865 (0.7)	790 (5.7)	
Emergency case (%)				
Elective	122,517 (93.6)	110,824 (94.8)	11,693 (83.9)	< 0.001
Emergency	8362 (6.4)	6111 (5.2)	2251 (16.1)	
General anaesthesia				
Yes	123,382 (94.3)	110,547 (94.5)	12,835 (92.0)	< 0.001
No	7497 (5.7)	6388 (5.5)	1109 (8.0)	
Surgery duration (median [IQR]), minutes	90.00 [50.00, 154.00]	90.00 [50.00, 150.00]	90.00 [45.00, 170.00]	0.765
Anesthesia duration (median [IQR]), minutes	144.00 [93.00, 221.00]	144.00 [94.00, 219.00]	146.00 [85.00, 246.00]	0.036
Comorbid diseases (%)				
Hypertension	22,644 (17.3)	19,310 (16.5)	3334 (23.9)	< 0.001
Ischemic heart disease	3373 (2.6)	2948 (2.5)	425 (3.0)	< 0.001
Diabetes	9379 (7.2)	8168 (7.0)	1211 (8.7)	< 0.001
COPD	5720 (4.4)	4994 (4.3)	726 (5.2)	< 0.001
Liver disease	22,891 (17.5)	20,212 (17.3)	2679 (19.2)	< 0.001
Renal failure	3661 (2.8)	2819 (2.4)	842 (6.0)	< 0.001
Malignancy tumor	40,391 (30.9)	36,846 (31.5)	3545 (25.4)	< 0.001
Surgical subspecialty (%)				
Central Nervous System	11,533 (8.8)	10,196 (8.7)	1337 (9.6)	< 0.001
Bones and Muscles	21,615 (16.5)	18,531 (15.8)	3084 (22.1)	
Pulmonary and Vascular	13,872 (10.6)	12,978 (11.1)	894 (6.4)	
Gastrointestinal Tract, Biliary Tract, Pancreas, and Liver	36,127 (27.6)	32,081 (27.4)	4046 (29.0)	
Breast, Thyroid, Otolaryngology, Head and Neck, Skin and Soft Tissue	25,007 (19.1)	23,054 (19.7)	1953 (14.0)	
Other	22,725 (17.4)	20,095 (17.2)	2630 (18.9)	
Preoperative laboratory tests (median [IQR])				
Hb, g/L	135.00 [123.00, 147.00]	135.00 [124.00, 147.00]	131.00 [113.00, 146.00]	< 0.001
WBC, × 10^9^/L	5.78 [4.77, 7.09]	5.72 [4.74, 6.95]	6.47 [5.08, 8.65]	< 0.001
NLR	2.09 [1.54, 3.05]	2.03 [1.51, 2.89]	2.91 [1.89, 5.45]	< 0.001
Na, mmol/L	141.20 [139.70, 142.70]	141.30 [139.80, 142.70]	140.90 [138.80, 142.60]	< 0.001
K, mmol/L	4.06 [3.84, 4.29]	4.06 [3.84, 4.28]	4.10 [3.82, 4.40]	< 0.001
BUN, mmol/L	4.90 [4.00, 6.00]	4.80 [4.00, 5.90]	5.10 [4.10, 6.60]	< 0.001
CRE, μmol/L	67.00 [57.00, 80.00]	67.00 [57.00, 80.00]	68.00 [57.00, 83.00]	< 0.001
Tbil, μmol/L	11.40 [8.60, 15.10]	11.30 [8.60, 14.90]	12.40 [9.00, 17.40]	< 0.001
ALB, g/L	44.10 [41.10, 46.80]	44.10 [41.30, 46.80]	43.40 [38.50, 47.00]	< 0.001
ALP, U/L	75.00 [61.00, 93.00]	74.00 [61.00, 92.00]	86.00 [68.00, 111.00]	< 0.001
ALT, U/L	18.00 [13.00, 28.00]	18.00 [13.00, 27.00]	24.00 [16.00, 41.00]	< 0.001
G, mmol/L	5.03 [4.65, 5.61]	5.01 [4.64, 5.55]	5.31 [4.78, 6.36]	< 0.001

*ALB* Albumin, *ALP* Alkaline Phosphatase, *ALT* Alanine Aminotransferase, *ASA-PS* American Society of Anesthesiologists Physical Status, *BMI* Body Mass Index, *BUN* Blood Urea Nitrogen, *CRE* Creatinine, *G* Blood Glucose, *Hb* Hemoglobin, *K* Potassium, *NLR* Neutrophil to Lymphocyte Ratio, *Na* Sodium, *SBP* Systolic Blood Pressure, *TBil* Total Bilirubin, *WBC* White Blood Cells.

### LDH and postoperative outcomes

**Figure 2:**
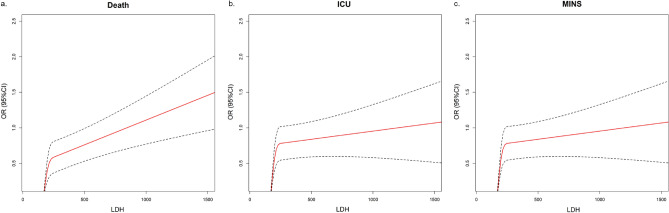
The restricted cubic spline of postoperative in-hospital mortality, ICU admission, and MINS after noncardiac surgery, according to preoperative LDH. Note: This figure presents the results of the restricted cubic spline analysis, depicting the relationship between preoperative LDH (Lactate Dehydrogenase) levels and three crucial postoperative outcomes: in-hospital mortality, ICU admission, and MINS (Myocardial Injury after Noncardiac Surgery). The x-axis represents the spectrum of preoperative LDH levels, capturing the linear patterns. The y-axis represents the odds ratios (OR) of outcomes. The curves showcase how the odds of each outcome change with varying LDH levels. The use of restricted cubic splines allows for a flexible representation of the relationship, capturing potential non-linear associations between LDH and postoperative outcomes.

As shown in Fig. 2, restricted spline fitting curves illustrates an escalating risk of postoperative mortality, ICU admission, and MINS with increasing preoperative LDH values In contrast to patients with normal preoperative LDH levels, those with high LDH (> 220 U/L) demonstrated high mortality rates (611 [4.382%] compared to 821 [0.702%]), an increased ICU admission rate (2093 [15.01%] versus 7500 [6.414%]), and a higher incidence of MINS (420 [3.012%] versus 648 [0.537%]). High LDH levels, when compared to the normal LDH group, maintained an independent and statistically significant association with heightened in-hospital mortality (OR 1.856, 95% CI 1.620–2.127; *P* < 0.001), ICU admission rate (OR 1.765, 95% CI 1.642–1.896; *P* < 0.001), and MINS (OR 1.911, 95% CI 1.643–2.223; *P* < 0.001), even after adjusting for confounding factors. Mortality, ICU admission, and MINS prediction outcomes with the IPTW method remained stable (OR 1.814, 95% CI 1.676–1.962; *P* < 0.001; OR 1.596, 95% CI 1.542–1.651; *P* < 0.001; OR 1.815, 95% CI 1.664–1.980; *P* < 0.001). Refer to Supplementary Table 1 for the matching details.

### Optimizing models with LDH extension

Additionally, we assessed the model performance for a number of conventional models, including the ASA, CCI, CARES, RCRI, and our multivariable logistic regression model, with and without the LDH element. Table 2 demonstrates that once LDH was included as a new marker, the AUROC values significantly improved. The Chi-square Statistics of the four models increased, with the exception of our multivariable logistic regression model. An improvement in classifying in models with LDH is shown by an increase in IDI or NRI. In all models using LDH, the Brier score dropped, indicating an improvement in the model's overall performance.

**Table 2:** Comparison of the discrimination and calibration for models with and without LDH, such as ASA, CCI, Ex-Care model, RCRI and our model.

	Discrimination		Calibration		Reclassification				Overall model performance
	AUROC	*P*-value	HL test (Chi-square statistic )	*P*-value	IDI	*P*-value	NRI	*P*-value	Brier score
ASA	0.836 (0.826–0.847)	Reference	26.656	0.001	Reference	Reference	Reference	Reference	0.0096
ASA plus LDH	0.861 (0.850–0.872)	< 0.001	36.295	< 0.001	0.021 (0.016–0.025)	< 0.001	0.164 (0.112–0.216)	< 0.001	0.0094
CCI	0.575 (0.562–0.589)	Reference	66.291	< 0.001	Reference	Reference	Reference	Reference	0.0108
CCI plus LDH	0.717 (0.702–0.732)	< 0.001	79.276	< 0.001	0.026 (0.021–0.031)	< 0.001	0.636 (0.586–0.687)	< 0.001	0.0107
Ex-care	0.875 (0.865–0.885)	Reference	61.823	< 0.001	Reference	Reference	Reference	Reference	0.0094
Ex-care plus LDH	0.881 (0.871–0.891)	< 0.001	22.328	0.004	0.018 (0.130–0.022)	< 0.001	0.130 (0.078–0.182)	< 0.001	0.0092
RCRI	0.555 (0.541–0.570)	Reference	31.886	< 0.001	Reference	Reference	Reference	Reference	0.3887
RCRI plus LDH	0.720 (0.704–0.735)	< 0.001	75.651	< 0.001	− 0.003 (− 0.012–0.005)	0.473	0.011 (0.003–0.019)	0.006	0.0106
Our Model without LDH	0.932 (0.926–0.939)	Reference	28.977	< 0.001	Reference	Reference	Reference	Reference	0.0089
Our Model with LDH	0.933 (0.927–0.940)	0.040	31.931	< 0.001	0.006 (0.003–0.008)	< 0.001	0.460 (0.409–0.512)	0.001	0.0088

*NRI* Net Reclassification Indices, *IDI* Integrated Discrimination Increment, *ASA* ASA Physical Status, *CCI* Charlson Comorbidity Index, *RCRI* Revised Cardiac Risk Index, *HL test* Hosmer–Lemeshow test.

### Sub-group analysis

After adjustment for all potential confounders listed in Table 1, preoperative LDH remained independent and significantly associated with increased mortality and morbidity. High LDH significantly influences the in-hospital mortality across different age groups, genders, ASA-PS classifications, surgical urgency levels, anesthesia methods, pre-existing comorbidities, and surgical sites. Preoperative high LDH led to increased mortality in patients with comorbidity. Although patients with high LDH had numerically higher mortality than patients with normal LDH in the group of patients with ischemic heart disease, the difference did not show statistical significance (see Table 3).

**Table 3:** OR (95%CI) for the association between the preoperative serum LDH level and in-hospital mortality, ICU admission, and MINS in all patients and subgroups.

Mortality	LDH < = 220	LDH > 220	*P*-Value
N (%)	821 (0.702%)	611 (4.382%)	
ORunadj	Reference	4.938 (4.447,5.482)	< 0.001
ORlrm-adj	Reference	1.856 (1.620,2.127)	< 0.001
ORIPTW-adj	Reference	1.814 (1.676,1.962)	< 0.001
Strata ORlrm-adj			
Sex			
Men (n = 66,561)	Reference	1.790 (1.507,2.125)	< 0.001
Women (n = 64,318)	Reference	1.918 (1.529,2.405)	< 0.001
Age			
14–60 (n = 91,352)	Reference	1.863 (1.546,2.243)	< 0.001
> 60 years (n = 39,527)	Reference	1.877 (1.532,2.301)	< 0.001
ASA-PS			
I–II (n = 96,406)	Reference	1.838 (1.263,2.675)	0.001
III (n = 32,818)	Reference	1.525 (1.195,1.944)	0.001
IV–V (1655)	Reference	2.029 (1.693,2.430)	< 0.001
Emergency case			
Emergency (n = 8362)	Reference	1.656 (1.387,1.976)	< 0.001
Elective (n = 122,517)	Reference	2.047 (1.661,2.522)	< 0.001
General anaesthesia			
Yes (n = 123,382)	Reference	1.827 (1.587,2.102)	< 0.001
No (n = 7497)	Reference	2.263 (1.222,4.192)	0.009
Comorbid diseases			
Hypertension			
Yes (n = 22,644)	Reference	1.705 (1.332,2.181)	< 0.001
No (n = 108,235)	Reference	1.895 (1.607,2.234)	< 0.001
Ischemic heart disease			
Yes (n = 3373)	Reference	1.444 (0.773,2.700)	0.249
No (n = 127,506)	Reference	1.890 (1.643,2.174)	< 0.001
Diabetes			
Yes (n = 9379)	Reference	1.523 (1.065,2.178)	0.021
No (n = 121,500)	Reference	1.907 (1.644,2.211)	< 0.001
COPD			
Yes (n = 5720)	Reference	2.322 (1.560,3.455)	< 0.001
No (n = 125,159)	Reference	1.789 (1.546,2.070)	< 0.001
Liver disease			
Yes (n = 22,891)	Reference	1.960 (1.502,2.556)	< 0.001
No (n = 107,988)	Reference	1.789 (1.524,2.100)	< 0.001
Renal failure			
Yes (n = 3661)	Reference	1.672 (1.025,2.726)	0.039
No (n = 127,218)	Reference	1.863 (1.615,2.149)	< 0.001
Malignancy tumor			
Yes (n = 40,391)	Reference	1.926 (1.448,2.562)	< 0.001
No (n = 90,488)	Reference	1.819 (1.557,2.127)	< 0.001
Surgery sites			
Central Nervous System (n = 11,533 )	Reference	1.770 (1.417,2.211)	< 0.001
Bones and Muscles (n = 21,615 )	Reference	1.171 (1.069,1.986)	0.020
Pulmonary and Vascular (n = 13,872 )	Reference	1.664 (1.092,3.025)	0.015
Gastrointestinal Tract, Biliary Tract, Pancreas, and Liver (n = 36,127 )	Reference	2.089 (1.635,2.669)	< 0.001
Breast, Thyroid, Otolaryngology, Head and Neck, Skin and Soft Tissue (n = 25,007 )	Reference	2.114 (1.097,4.589)	0.015

ICU admission	LDH < = 220	LDH > 220	*P*-Value
N (%)	7500 (6.414%)	2093 (15.010%)	
ORunadj	Reference	2.577 (2.446,2.715)	< 0.001
ORlrm-adj	Reference	1.765 (1.642,1.896)	< 0.001
ORIPTW-adj	Reference	1.596 (1.542,1.651)	< 0.001

MINS	LDH < = 220	LDH > 220	*P*-Value
N (%)	628 (0.537%)	420 (3.012%)	
ORunadj	Reference	5.752 (5.077,6.516)	< 0.001
ORlrm-adj	Reference	1.911 (1.643,2.223)	< 0.001
ORIPTW-adj	Reference	1.815 (1.664,1.98)	< 0.001

*OR* Odds ratios.

Note: Unadjusted Model (OR_unadj_): The unadjusted odds ratio was calculated using a logistic regression model without additional covariate adjustments. Adjusted Logistic Regression Model (OR_lrm-adj_): Odds ratios were adjusted for relevant covariates using a multivariable logistic regression model. IPTW-Assisted Adjusted Model (OR_IPTW-adj_): Inverse Probability of Treatment Weighting (IPTW) was applied to adjust for treatment selection bias, and odds ratios were estimated using a multivariable logistic regression model. Subgroup analyses were performed to assess the relationship between LDH and mortality outcomes within various subpopulations.

### Sensitivity analysis

In the sensitivity analysis excluding patients under 18 years, we reassessed the relationship between preoperative LDH levels and prognosis. The age criteria for this analysis included individuals aged 18 and above (n = 128,033). The results indicated a significant association between higher preoperative LDH levels and in-hospital mortality (4.401% vs. 0.707%; 1.852, 95% CI 1.614–2.125, *P* < 0.001), myocardial injury after noncardiac surgery (MINS) (3.027% vs. 0.537%; OR 1.896, 95% CI 1.627–2.208, *P* < 0.001), and ICU admission (15.061% vs. 6.468%; OR 1.749, 95% CI 1.627–1.881, *P* < 0.001), consistent with the main analysis. This supports the robustness and generalizability of our research findings across different age groups.

## Discussion

Our multivariable logistic regression study revealed a significant relationship between preoperative LDH levels and postoperative mortality and morbidity following noncardiac surgery. In addition, propensity score weighting method returned unchanged results, which again validated the robustness of the findings.

In subgroup analysis, increased LDH was not significantly associated with increased mortality in the ischemic heart disease subgroup (OR 1.444, 95% CI 0.773–2.700; *P* = 0.249), which may be related to the general increase of LDH level caused by myocardial injury before surgery. The low AUROC of LDH for predicting ICU admission may be due to the fact that ICU retention is often affected by many factors such as social environment and patient condition. This complexity of the results makes the predictive performance of LDH in this scenario challenging. The identification, correction, reclassification, and overall model performance of both the traditional model and our model could be improved to varying degrees by adding LDH. Thus, we can conservatively conclude that high preoperative LDH levels are associated with increased in-hospital mortality, MINS, and ICU admission risk after noncardiac surgery.

The predictive significance of preoperative blood LDH concentration for postoperative adverse events has only been examined in a small number of trials, and it is uncertain whether this correlation could extend to other surgical procedures.^7–9^. It was discovered that pulmonary problems following thoracic surgery were predicted by the preoperative high LDH level (> 230 U/l)^8^. Mitsudomi et al.^19^ found that preoperative LDH levels greater than 178U/L were significantly associated with mortality after pneumonectomy (n = 62). Ruoyu Zhang^9^ in 2019 analyzed LDH as a continuous indicator for the first time and demonstrated its linear relationship with a predictor of pulmonary complications. A review in 2019^20^ summarizes the recent advances in the design and development of inhibitors, pointing out their specificity and therapeutic potential. This study indicates that high preoperative LDH is a significant predictor of postoperative mortality, ICU stay, and MINS. In future related predictive models, it may be beneficial to incorporate LDH as a predictor for simultaneous modeling. As LDH is a readily available preoperative laboratory indicator and a modifiable factor, prospective research in the future is warranted to explore its causal relationship with postoperative outcomes, aiming to gain a better understanding of its role in the disease process. This exploration will contribute to determining whether LDH can be a target for intervention to improve patient postoperative outcomes.

This study investigated the relationships among different surgical subgroups, excluding thoracic surgery, and concurrently expanded the utility of LDH in predicting surgical outcomes. To mitigate the impact of data imbalance, we employed propensity score weighting combined with a multifactor logistic regression model. Furthermore, leveraging the advantage of sample size, we depicted a restricted cubic spline function curve to elaborate on the nonlinear relationship between LDH and postoperative mortality. Beyond that, we demonstrated the additional contribution of LDH to various commonly used surgical prognostic models by incorporating LDH as an extended variable.

We have several limitations that should be noted. First, there is selection bias as a result of the use of retrospective data from a single center. Second, some unadjusted variables, including ECG, myocardial enzyme, the effects of surgery and perioperative treatment, may still result in residual confounding factor even when we employ multi-factor logistic regression and the propensity score weighting technique. Third, it is essential to acknowledge that this study is purely observational in nature. As such, while it identifies associations between variables, it does not establish causality. The findings should be interpreted with caution, recognizing that inherent limitations in observational research preclude the attribution of causal relationships. Future prospective studies or randomized controlled trials are warranted to further explore the causative aspects of the observed associations. Finally, in this study, the definition of MINS did not include measuring hs-cTnT in all patients. This may result in some cases of MINS being missed. However, it is important to note that this study aims to unfold within the context of real-world practice, where universally measuring hs-cTnT may not always be feasible or practical in the clinical environment. While this is one of the limitations of the study, we emphasize that our goal is to provide valuable information in actual clinical settings and to support physicians in making decisions in resource-constrained environments.

## Conclusion

After noncardiac surgery, greater preoperative LDH levels were linked to an increased risk of in-hospital mortality, MINS, and ICU admission. LDH could provide extra predictive information in addition to the commonly used surgical prognostic scores, including the ASA, CCI, CARES and RCRI models.

### Supplementary Information

Supplementary Table 1.

## Data Availability

The datasets presented in this article are not readily available because this dataset was not publicly available due to ethics committee requirements. Requests to access the datasets should be directed to 739501155@qq.com.
